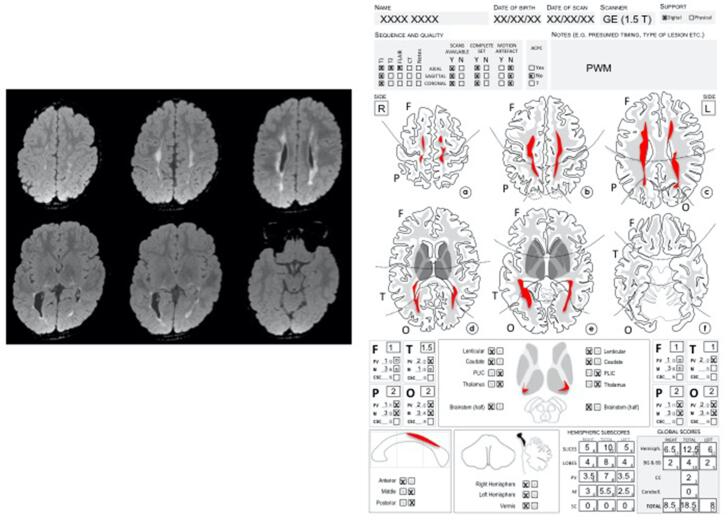# Structural brain damage and visual disorders in children with cerebral palsy due to periventricular leukomalacia

**DOI:** 10.1016/j.nicl.2020.102430

**Published:** 2020-09-11

**Authors:** Francesca Tinelli, Andrea Guzzetta, Giulia Purpura, Rosa Pasquariello, Giovanni Cioni, Simona Fiori

**Affiliations:** a Department of Developmental Neuroscience, IRCCS Fondazione Stella Maris, Viale del Tirreno 331, Calambrone, Pisa, Italy; b Department of Clinical and Experimental Medicine, University of Pisa, Via Savi 10, Pisa, Italy

**Keywords:** PVL, Periventricular Leukomalacia, MRI, Magnetic Resonance Imaging, CP, Cerebral Palsy, VTS, Visual Total Score, Cerebral visual impairment, Visual function, Brain magnetic resonance imaging, Cerebral palsy, Children, Periventricular leukomalacia

## Abstract

**Aim:**

To systematically explore the relationship between type and severity of brain lesion on Magnetic Resonance Imaging (MRI) and visual function in a large cohort of children with periventricular leukomalacia (PVL).

**Methods:**

94 children with bilateral cerebral palsy (CP) and history of PVL were recruited at Stella Maris Scientific Institute in Pisa (Italy). We included data of participants (72) with at least one MRI after the age of three years and an evaluation of visual function including fixation, following, saccades, nystagmus, acuity, visual field, stereopsis and color perception.

Brain lesions location and extent were assessed by a semi-quantitative MRI-scale for children with CP.

**Results:**

Brain lesion severity strongly correlated with visual function total score (global MRI score **p = .000;** hemispheric score **p = .001** and subcortical score **p = .000**). Moreover, visual acuity, visual field, stereopsis and colour were compromised when a cortical damage was present, while ocular motricity (and in particular fixation and saccades) were compromised in presence of subcortical brain damage.

**Interpretation:**

Structural MRI is valuable for understanding the relationship between brain lesion severity and visual function in children with CP.

## Highlights

• There is a strong correlation between brain lesion severity and visual function, evident also with a Structural MRI.
• It is confirmed the validity of MRI semi-quantitative scale published by Fiori et al. (2014).
• There is a frequent association of PVL with thalamic lesions with important repercussion on visual function.

## 1 Introduction

Children with CP very often have visual perceptual disorders including those that involve the dysfunction of anterior, posterior, or a combination of both visual pathways (Dutton and Jacobson, 2001, Good et al., 1994, Good et al., 2001; Schenk-Rootlieb et al., 1994). Due to their relevance, visual perceptual disorders are considered a core symptom of CP, rather than an associated symptom (Rosenbaum et al., 2007).

Cerebral Visual Impairment (CVI) is a major cause of low vision worldwide, and about 60–70% of children with CP manifest it (Schenk-Rootlieb et al., 1994). CVI causes include the posterior visual pathways dysfunction (including optic radiations, occipital cortex, visual associative areas). Its reported prevalence in the CP population varies greatly among studies, very often depending on the selection criteria (population-based studies, baseline data for clinical trials, and the like) and on the source of clinical information (registers, hospital record, direct testing) (Guzzetta, 2014). It is not surprising that the incidence of CVI within the CP population is so high, since brain areas whose injury determines the motor deficit resulting in CP are anatomically closely located to the distributed network of brain areas responsible for visual perception (Schenk-Rootlieb et al., 1994).

Periventricular leukomalacia (PVL) is the lesion that most clearly illustrates this relationship between brain damage, CP and CVI, given that it is known to involve both the corticospinal tract and the visual pathways, mostly including optic tracts, posterior thalamus and optic radiations (Jacobson and Dutton, 2000, Lanzi et al., 1998). In children with PVL, posterior visual pathways dysfunction manifests through visual field abnormalities, reduced visual acuity, refractive errors, altered contrast sensitivity, abnormal stereopsis and optokinetic nystagmus. However, impairment of the oculomotor system is also typical in children with PVL, determining strabismus, abnormal fixation, following and saccadic movements disorder. Finally, PVL may also determine a typical fundus oculi appearance with a pale, cupped optic disc, reflecting an atypical form of secondary optic nerve hypoplasia (Ruberto et al., 2006).

In recent years, there has been an increased interest in the understanding of the critical areas for the development of visual function, which seems to depend on the integrity of an enlarged network that includes not only optic radiations and the primary visual cortex but also other cortical and subcortical areas, such as the frontal or temporal lobes or basal ganglia (Ramenghi et al., 2010). However, despite its frequency and clinical relevance, there are few reports, generally including small samples, about the relationship between visual dysfunction and brain lesion characteristics in children with CP due to PVL (Uggetti et al., 1996). Furthermore, there has been increasing evidence that the thalami are often primarily or secondarily affected in children with PVL (Lin et al., 2001) and that they play a role in the development of visual function (Rushmore et al., 2005). In this sense, congenital brain lesions are a fascinating model to study the relationship between brain structure and function through dysfunction.

The purposes of the present study were: a) to explore the relationship between brain lesion severity, and visual function in a large sample of children with PVL; b) to define the possible role of specific brain areas and structures in fixation, following, saccades, nystagmus, visual acuity, visual field, stereopsis and colour perception in the same group of children with PVL.

## 2 Methods

### 2.1 Participants

Participants were recruited at the IRCCS Stella Maris Foundation in Pisa, Italy, a research hospital devoted to neurodevelopmental disability. Children who had done at least one MRI after three years of age and a comprehensive visual assessment were considered eligible. The study was approved by the Ethics Committee of the Meyer’s Hospital. Informed parental consent was obtained for all participants.

### 2.2 Procedure

All children underwent an assessment of visual function at the vision laboratory of the hospital. Children were assessed by a well-trained developmental therapist (G.P.) and a paediatric neurologist (F.T.), routinely involved in the clinical evaluation of children with CP and visual disorders. Brain lesions were scored using a semi-quantitative MRI-scale (sqMRI scale, (Fiori et al., 2014, Fiori et al., 2015). The scale was applied by a child neurologist (S.F.) supported by a neuroradiologist (R.P.) when needed (Fiori et al., 2014). Both MRI raters were blinded to the outcome of the visual assessment.

### 2.3 Visual score

All children underwent a battery of age-specific tests assessing visual function including fixation, following, saccades, nystagmus, visual acuity, visual field, stereopsis and colour perception.

Fixation was tested observing the ability of the child to fix on a black/white or coloured target; Following was tested observing the ability of the child to follow a coloured target horizontally, vertically and in a full circle.

Saccades were tested observing the ability of the child to move quickly his/her eyes from a target to another one;

Nystagmus was tested observing children’s eyes. It is a condition in which the eyes move rapidly and uncontrollably;

Acuity was assessed binocularly by means of the Teller acuity card procedure (Teller et al., 1986). This method is based on an inborn preference for a pattern (black and white gratings of decreasing stripe widths depicted on cards) over a uniform field. The threshold of acuity is taken as the minimum stripe width to which the subject consistently responds. Acuity values were compared to age-specific normative data reported in the literature (van Hof-van Duin et al., 1992). A result within 2 standard deviation was considered normal.

Binocular visual fields were assessed using kinetic perimetry, according to the technique described in detail by van Hof-van Duin (van Hof-van Duin et al., 1992). The apparatus consists of two 4-cm wide black metal strips, mounted perpendicularly to each other and bent to form 2 arcs, each with a radius of 40 cm. The child is held sitting or lying in the centre of the arc perimeter, with the chin supported. During central fixation of a 6° diameter white ball, an identical target is moved from the periphery towards the fixation point, along one of the arcs of the perimeter, at a velocity of about 3°/s. Eye and head movements towards the peripheral ball are used to estimate the outline of the visual fields. Age-specific normative data are reported in the literature (Wilson et al., 1991).

Stereopsis is the highest form of binocular coordination that can be assessed (Afsari et al., 2013). In this study it was evaluated by means of Frisby Stereopsis Screening Test (Frisby et al., 1996). Briefly, the participant’s task is to detect a circle containing a pattern of geometric objects (target) visible within a mosaic of similar geometric shapes. The target and background are printed on opposite sides of a Perspex plate, and so differ in physical depth. The Frisby test comprises three plates, each of which can be presented at one of several different possible distances to obtain a range of disparities. A positive result is recorded if the subject’s scanning eye movements stop consistently at the correct target upon repeated testing. Stereopsis values are expressed in sec/arc. Participants who could not identify target at 600 sec/arc were classified as stereo-negative.

Colour perception was evaluated by means of Color Vision Test Plates for Infants. The child has to recognize the picture made by different coloured circles and if he/she is not able to talk, he/she can indicate with the hand or eyes in which part of the book is the picture that the observer tells him.

For each item it is possible to give a score of 0 if it is not compromised or 1 when there is an impairment. A visual total score (VTS) was obtained from the sum of all of the items, ranging from 0 to 8.

See Table in Supplementary Materials for a detailed description of visual severity scores in the population.

### 2.4 MRI assessment

**Supplementary Fig. 1:**
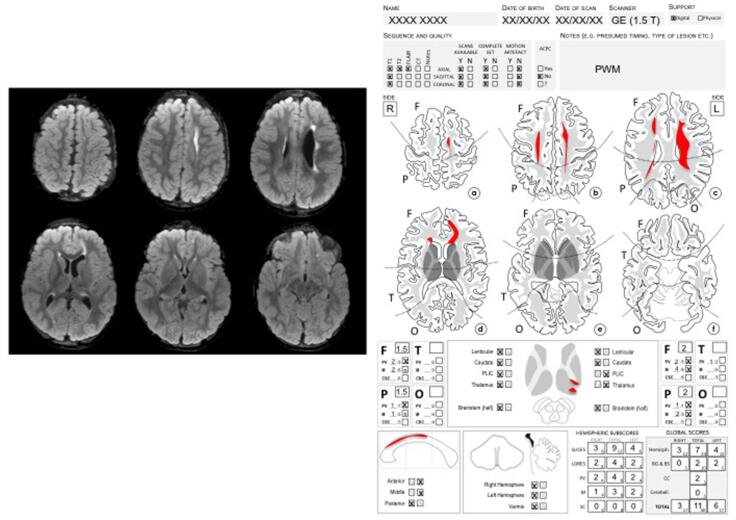


MRIs were classified according to a previously described reliable and validated semi-quantitative scale for assessing brain lesion severity in children with cerebral palsy (Fiori et al., 2014). According to the scoring procedure described by by Fiori and collaborators (Fiori et al., 2014), brain lesion is graphically represented onto a six-axial-slices template. Raw scores for each lobe, subcortical structures (basal ganglia, thalamus, posterior limb of internal capsule (PLIC) and brainstem), corpus callosum and cerebellum are calculated. The scoring procedure results in summary scores for right and left hemisphere: lobar score (frontal, parietal, temporal, occipital, bilateral maximum score of 6 for each lobe); hemispheric score (the summary of frontal, parietal, temporal and occipital scores, bilateral maximum score of 24); subcortical score (the summary of lenticular, caudate, PLIC, thalamus, and brainstem scores, bilateral maximum score of 10); global score (hemispheric summary score on both side plus corpus callosum and cerebellum scores, maximum score of 40), with higher score representing more severe pathology. For the purposes of this study, all scores were calculated as bilateral. See Table in Supplementary Materials for a detailed description of brain lesion severity scores in the population. Examples of the scoring sheets are provided in Supplementary Figs. 1 and 2.

## 3 Statistical analyses

The hemispheric score, the subcortical score, the global score and the visual total score firstly were included in the correlation analysis; we further explored correlations between single lobes (frontal, temporal, parietal and occipital) and visual total score. For correlation analyses, we used the Spearman (rho) correlation coefficient and we evaluated statistical significance considering Bonferroni adjustement of p-value to account for multiple comparisons, thus setting significance level for those comparisons for p-value < 0.007. The relationships between single subcortical structures (lenticular, caudate, PLIC, thalamus, and brainstem scores) and visual total score, corpus callosum and cerebellum and visual total score were also explored.

Moreover, each item of the visual assessment was included separately in the analysis, to check for differences in brain lesion site and severity underlying the item dysfunction (normal/abnormal).

In order to better understand the role of brain lesion site on each visual function item, a between group *t*-test was performed by splitting the sample according to the abnormality of one specific function (i.e. those subjects with score 0 or 1 at that specific item). Mean and standard deviation (SD) were used to describe Global MRI score and Hemispheric score while student independent *T*-test was used for comparisons between groups. Subcortical scores were reported as median and 25°-75° percentile because of their skewed distribution and comparisons between groups were performed using Mann-Whitney *U* test (see Table 1).

**Table 1:** Comparison of MRI scores between subjects with normal (N) and abnormal (A) scores for each visual item.

Different Items	Evaluation (subjects Numerosity)	Global Score (Mean ± SD)	p value*	Hemispheric Score (Mean ± SD)	p value*	Subcortical Score (Median [25°Pc-75° Pc])	p value#
**Fixation**	N (21)	12.81 ± 4.77	**0.0128**	10.43 ± 3.36	0.075	0 [0–0]	**0.0011**
	A (51)	16.31 ± 5.48		12.08 ± 3.58		2 [0–4]	

**Following**	N (16)	12.47 ± 4.69	**0.0186**	10.09 ± 3.34	0.056	0 [0–2]	**0.0103**
	A (56)	16.10 ± 5.47		12.03 ± 3.55		2 [0–4]	

**Saccades**	N (14)	12.25 ± 5.02	**0.0198**	10.18 ± 3.45	0.0985	0 [0–0]	**0.0051**
	A (58)	16.03 ± 5.38		11.94 ± 3.55		2 [0–4]	

**Nystagmus**	N (42)	15.04 ± 5.92	0.643	11.58 ± 3.91	0.969	1 [0–4]	0.267
	A (30)	15.65 ± 4.90		11.62 ± 3.11		2 [0–4]	

**Acuity**	N (25)	12.52 ± 5.05	**0.0013**	9.96 ± 3.30	**0.0039**	0 [0–1]	**0.0013**
	A (47)	16.77 ± 5.17		12.47 ± 3.44		2 [0–4]	

**V Fields**	N (44)	13.77 ± 5.11	**0.0026**	10.64 ± 3.36	**0.0036**	0 [0–4]	**0.0071**
	A (28)	17.68 ± 5.29		13.11 ± 3.44		4 [1.5–4]	

**Stereopsis**	N (19)	12.32 ± 5.45	**0.0051**	9.74 ± 3.62	**0.0073**	0 [0–4]	**0.0196**
	A (53)	16.36 ± 5.15		12.26 ± 3.35		2 [0–4]	

**Colour**	N (41)	13.28 ± 4.88	**0.0002**	10.22 ± 3.07	**0.0001**	0 [0–4]	**0.0108**
	A (31)	17.95 ± 5.16		13.44 ± 3.40		4 [0–4]	

*t.test; # Mann-Withney test.

Correlation between gestational age and lesion (hemispheric score, the subcortical score, the global score) and the visual total score was evaluated using the Spearman (rs) correlation coefficient. In the same way we analyzed the correlation between age at test and visual total score.

P-value < 0.05 was considered to be statistically significant.

## 4 Results

### 4.1 Demographics

Ninety-four children (57 males, 37 females) with CP and a brain MRI indicating a PVL were recruited. One subject was excluded because of complete blindness for a prematurity-related stage III retinopathy, and 21 other subjects were excluded because of the lack of a visual assessment within 1 year from MRI exam. This is a retrospective study and it can explain the high percentage of missed subjects (more than 23%).

The final sample consisted of 72 children with cerebral palsy (42 males and 30 females), mean gestational age 32.4 weeks (range 24–40; SD 4.6 weeks), mean age at visual assessment 5.6 years (range 3.2–14.4 yrs; SD 3.4 yrs), mean age at MRI 5.8 years (range 3–14.4 yrs; SD 3.7 yrs). We kept in consideration the visual assessment done at the same age of MRI or the nearest to the MRI exam. About Gross Motor Function Classification System, subjects were distributed in this way: I = 7; II = 21; III = 20; IV = 17; V = 4; unknown = 3. Fifteen subjects (21%) had no visual disorder, 4 of them (5%) had solely a peripheral visual disorder, 15 subjects (21%) had a cerebral visual disorder and 38 subjects (53%) had a mixed visual disorder.

### 4.2 Visual assessment

**Fig. 1:**
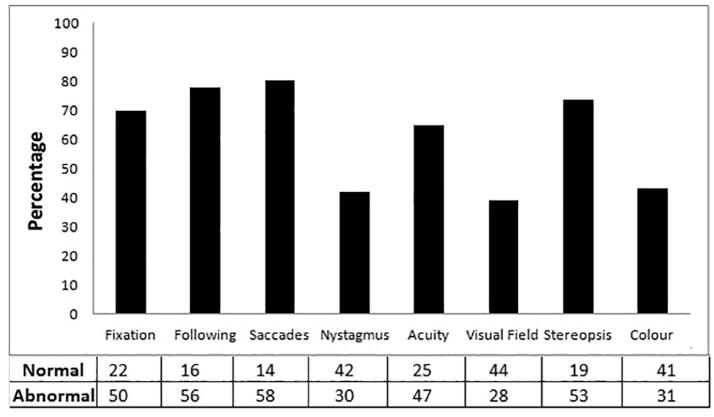
Incidence of visual disorders in PVL children.

At the visual assessment only 5 subjects (6,9%) reported visual total score 0 that is they had no visual problem, 2 scored 1 (3%), 6 scored 2 (8,3%), 10 scored 3 (13,8%), 5 scored 4 (6,9%), 7 scored 5 (9,7%), 14 scored 6 (19,5%), 13 scored 7 (18,1%), 10 scored 8 (13,8%). Percentages of children with problems in fixation, following, saccades, nystagmus, visual acuity, visual field stereopsis and color were reported in Fig. 1. Number of subjects with normal or abnormal score in each function was reported in the panel under the figure.

### 4.3 Correlation between visual total score and MRI scores

**Fig. 2:**
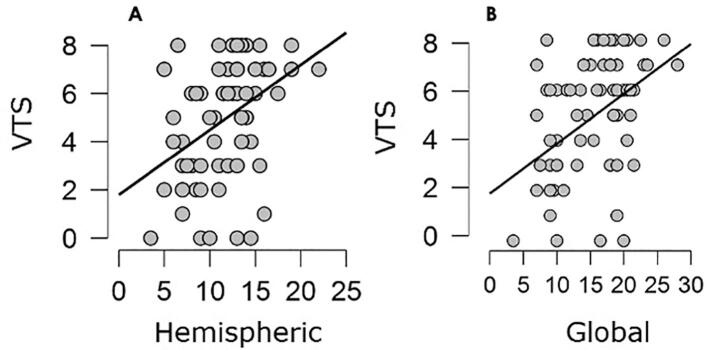
Correlation between Visual Total Score and Hemispheric and Global MRI score.

A highly significant correlation was found between VTS and global MRI score (**p = .000; rho = 0.414,** see Fig. 2), hemispheric score (**p = .001; rho = 0.387**, see Fig. 2 B) and subcortical score (**p = .000; rho = 0.470**).

### 4.4 Correlation between visual total score and single lobe scores

Concerning *cortical lesion location* damage, occipital lobes damage positively correlated with the VTS (**p = .000; rho = 0.443**), while no significant correlation was found with frontal, temporal and parietal lobes involvement.

### 4.5 Correlation between visual total score and single subcortical structures

Concerning *subcortical structures* damage, PLICs involvement has a tendency to correlate with VTS (**p = .008; rho = 0.31)** while Thalami involvement positively correlated with VTS (**p = .000; rho = 0.46**), while no significant correlation was found with lenticular, caudate and brainstem scores.

### 4.6 Correlation between visual total score and corpus callosum and cerebellum

No correlation was found neither between corpus callosum and VTS, nor between cerebellum and VTS.

### 4.7 Comparison of MRI scores between subjects with normal and abnormal scores for each visual item

Global MRI score mean values resulted significantly higher in children with an impairment in all items except for nystagmus (see p-values in Tab. 1). Hemispheric severity scores were significantly higher (corresponding to more severe lesions) in children with impaired visual acuity, visual field, stereopsis and colour perception (see p values in Tab. 1). Subcortical score means values resulted significantly higher in children with an impairment in all the visual items except for nystagmus (see p values in Table 1).

### 4.8 Correlation between gestational age and lesion and VTS and age and VTS

We found no correlation between gestational age and lesion and between gestational age and Visual Total Score and age and Visual total score.

## 5 Discussion

To our knowledge this is the first study that correlates brain lesions severity and visual function impairment in a large sample of children with PVL.

First of all, we found that the most compromised visual items in our sample were ocular motricity (including fixation 71%, following 77.7% and saccades 80.5%), visual acuity (65.3%) and stereopsis (74%). These results are perfectly in agreement with previously published literature and the most recent paper by Fazzi and colleagues (Fazzi et al., 2012), where the authors found an impairment in particular of saccadic movements and visual acuity in a sample of 129 children with cerebral palsy.

The semi-quantitative MRI scale for assessing brain lesions severity demonstrated a relationship with visual function measures in children with CP due to PVL. The three summary scores (global, hemispheric and subcortical) of the semi-quantitative brain MRI scale positively correlated with visual total score in a highly significant statistical way. It means that children with higher severity of brain lesion have higher severity of visual dysfunction.

Further, by considering single lobes, only occipital lobes’ lesion severity correlated with visual total score. Occipital cortex is indeed the place where primary visual cortex (V1) is localized. In primates, including humans, the perception of visual information is mediated by a pathway from retina to primary visual cortex (V1, striate cortex) via the lateral geniculate nucleus (LGN) of the thalamus and optic radiations (Felleman and Van Essen, 1991). V1 contains a detailed map of the whole visual field and is the first station in which binocular cells are present and, in the macaque monkey, strong orientation and direction selectivity cells were also described (Hubel and Wiesel, 1968). Moreover, during evolution, the expansion of primary visual cortex is associated with an increase in visual acuity (Mazade and Alonso, 2017). From V1, the visual information is then distributed to extrastriate cortical areas following two parallel pathways: the dorsal visual stream, which progresses to the parietal cortex via the middle temporal area (MT) and mediates visually guided behaviors; and the ventral visual stream, which reaches the temporal cortex via areas V2, V3 and V4, and mediates objects perception (Goodale and Milner, 1992).

On the other side, among subcortical structures a strong positive correlation with visual total score was found with thalami severity scores and a tendency with PLIC. In the visual system the LGN of the dorsal thalamus is the gateway through which information reaches the cerebral cortex. The thalamus is a nexus connecting the subcortical and cortical oculomotor centres that orchestrate the coordination of voluntary and reflexive eye movements necessary for coherent visually guided behaviour. An oculomotor function for the central and posterolateral thalamus has been suggested by animal studies showing that saccades can be elicited by electrical stimulation of thalamic nuclei and that single units in them are active in relation to saccades (Schlag-Rey and Schlag, 1984). Abnormalities of voluntary and visually triggered saccades have been reported in patients with acute thalamic lesions, even if recently Rafal et al. (2014) demonstrated that the thalamus is involved in the control of fixation for visually triggered, but not for voluntary saccades.

Moreover, the link between thalamus and PLIC can be easily understood since that the retrogeniculate part of the internal capsula contains fibers from the optic system, coming from the LGN of the thalamus and more posteriorly it becomes the optic radiation.

In light of these premises, the results of the comparison of MRI scores and single items of visual function (normal or impaired) are logical. It is of great interest to analyze the differences we found between the hemispheric and the subcortical scores.

The comparison between hemispheric score was statistically significant only for visual acuity, visual field, stereopsis and colour, indicating these functions might recruit primary visual cortex. Instead, when we considered the subcortical scores, a statistically difference was found for all visual items except for nystagmus but all subjects with subcortical impairment have also hemispheric compromission (see Table in Supplementary Material). What subcortical compromission seems to add is the important disorder in fixation, following and saccades. Therefore, once again the importance of subcortical structures integrity for the function of ocular motricity was confirmed.

These results are in agreement with those reported by Ricci and colleagues (Ricci et al., 2006) where 6 of the 12 subjects with clear indications of atrophy of the thalami had severe and wide-ranging abnormalities of visual function in all testing domains i.e. ocular movements, acuity, visual field and fixation shift.

What is clear now from literature is that thalamic involvement commonly accompanies PVL and is commensurate with the extent of white matter lesions (Lin et al., 2001). At the present, however, it is difficult to clarify the pathogenesis of thalamic lesions in infants with PVL. Some years ago Lin and colleagues (Lin et al., 2001) suggested that the intrinsic vulnerability is a probable factor related to the thalamic involvement. Metabolic demands of the thalami are considered to be higher than those of the white matter. Thus, the thalami are readily damaged by hypoxic–ischemic injury. Growth restriction was proposed as another explanation. Severe white-matter damage is likely to influence growth of the brain structures, which have some connection to the damaged white matter. Axonal damage in the cerebral hemisphere, which manifests as PVL may reduce growth of the thalamus. Kerbergen and colleagues (Kersbergen et al., 2015) sustained, instead, that it is the white matter damage in cystic-PVL that leads to axonal disturbances, which subsequently affect thalamic development. Impaired maturation of the late oligodendrocyte progenitors, known to be especially susceptible to ischemic damage, leads to a failure in myelination (Back and Miller, 2014). Afferent and efferent axons between the thalamus and cortex as well as the thalamus and the brain stem and cerebellum may be affected, thereby, impairing normal development of the connections that are being formed during the last trimester of gestation (Volpe, 2009). Alternatively, neuronal loss and gliosis may directly influence thalamic atrophy because of impaired input to the thalamus.

In conclusion, this study demonstrates that visual disorders in children with PVL correlate with the severity of brain lesion assessed by a semi-quantitative MRI scale and that visual acuity, visual field, stereopsis and colour impairment seem to be linked to cortical damage, while ocular motricity disorders are strictly linked to subcortical damage.

## CRediT authorship contribution statement

**Francesca Tinelli:** Conceptualization, Data curation, Formal analysis, Methodology, Writing - original draft, Writing - review & editing. **Andrea Guzzetta:** Methodology, Supervision. **Giulia Purpura:** Data curationValidation. **Rosa Pasquariello:** Data curation, Validation. **Giovanni Cioni:** Supervision. **Simona Fiori:** Conceptualization, Data curation, Formal analysis, Methodology, Writing - original draft, Writing - review & editing.

## Figures and Tables

**Supplementary Fig. 2:**