# Supplementary material for: Structural brain damage and visual disorders in children with cerebral palsy due to periventricular leukomalacia
Source: Neuroimage Clin. 2020 Sep 11;28:102430. doi: 10.1016/j.nicl.2020.102430 (PMC7519396; doi:10.1016/j.nicl.2020.102430)
Supplement: Supplementary Data 1 [file mmc1.docx]

|  |  |  | **MRI Score** | | | **Visual Assessment** | | | | | | | | |
| --- | --- | --- | --- | --- | --- | --- | --- | --- | --- | --- | --- | --- | --- | --- |
| ***Subject*** | ***MRI***  ***(years)*** | ***GA*** | ***H score*** | ***S score*** | ***G score*** | ***Fixation*** | ***Following*** | ***Saccades*** | ***Nystagmus*** | ***V. Acuity*** | ***V. field*** | ***Stereopsis*** | ***Colour*** | **VTS** |
| **1** | 11,5 | 40 | 7 | 2 | 10 | 1 | 0 | 0 | 0 | 1 | 0 | 1 | 0 | 3 |
| **2** | 3 | 30 | 12 | 0 | 13 | 1 | 0 | 0 | 1 | 0 | 0 | 1 | 0 | 3 |
| **3** | 5,1 | 26 | 6 | 0 | 7 | 1 | 1 | 1 | 0 | 1 | 1 | 0 | 0 | 5 |
| **4** | 4,6 | 31 | 14 | 4 | 19 | 0 | 1 | 1 | 0 | 1 | 1 | 1 | 0 | 5 |
| **5** | 3,4 | 24 | 5 | 0 | 7 | 1 | 1 | 1 | 1 | 1 | 0 | 1 | 1 | 7 |
| **6** | 3,3 | 33 | 8 | 0 | 9 | 1 | 1 | 0 | 0 | 0 | 0 | 1 | 0 | 3 |
| **7** | 3,2 | 37 | 15,5 | 4 | 22,5 | 1 | 1 | 1 | 1 | 1 | 1 | 1 | 1 | 8 |
| **8** | 3 | 30 | 8,5 | 0 | 9,5 | 1 | 0 | 0 | 0 | 0 | 0 | 1 | 0 | 2 |
| **9** | 3,2 | 30 | 9 | 0 | 10 | 0 | 1 | 1 | 0 | 1 | 1 | 1 | 1 | 6 |
| **10** | 5,8 | 31 | 7,5 | 0 | 7,5 | 1 | 1 | 1 | 0 | 0 | 0 | 0 | 0 | 3 |
| **11** | 8 | 24 | 22 | 4 | 28 | 1 | 1 | 1 | 0 | 1 | 1 | 1 | 1 | 7 |
| **12** | 3,2 | 28 | 9 | 0 | 10 | 0 | 0 | 0 | 0 | 0 | 0 | 0 | 0 | 0 |
| **13** | 4,4 | 29 | 11,5 | 7 | 21,5 | 1 | 1 | 1 | 1 | 1 | 0 | 1 | 0 | 6 |
| **14** | 6 | 33 | 12,5 | 4 | 18,5 | 1 | 1 | 1 | 1 | 1 | 0 | 1 | 0 | 6 |
| **15** | 3,5 | 39 | 11 | 0 | 11 | 0 | 0 | 0 | 1 | 0 | 0 | 0 | 1 | 2 |
| **16** | 5,9 | 40 | 13 | 4 | 18 | 1 | 1 | 1 | 1 | 1 | 0 | 1 | 1 | 7 |
| **17** | 9 | 32 | 8,5 | 0 | 8,5 | 1 | 1 | 1 | 1 | 1 | 0 | 1 | 0 | 6 |
| **18** | 3 | 30 | 13 | 2 | 16 | 1 | 1 | 1 | 1 | 1 | 1 | 1 | 1 | 8 |
| **19** | 1,1 | 38 | 14 | 4 | 21 | 1 | 1 | 0 | 0 | 1 | 1 | 1 | 0 | 5 |
| **20** | 3,2 | 31 | 9 | 0 | 10 | 1 | 1 | 0 | 0 | 0 | 0 | 1 | 0 | 3 |
| **21** | 3,7 | 40 | 11 | 4 | 18 | 1 | 1 | 1 | 0 | 0 | 0 | 0 | 0 | 3 |
| **22** | 2,9 | 31 | 14,5 | 0 | 15,5 | 0 | 1 | 1 | 0 | 1 | 0 | 1 | 0 | 4 |
| **23** | 3,3 | 37 | 11,5 | 2 | 16,5 | 1 | 1 | 1 | 1 | 1 | 0 | 1 | 0 | 6 |
| **24** | 3 | 35 | 12 | 4 | 18 | 1 | 1 | 1 | 0 | 1 | 1 | 1 | 1 | 7 |
| **25** | 3 | 28 | 12,5 | 0 | 13,5 | 1 | 1 | 1 | 0 | 1 | 1 | 1 | 0 | 6 |
| **26** | 12,7 | 38 | 13,5 | 2 | 18,5 | 1 | 1 | 1 | 0 | 0 | 0 | 1 | 1 | 5 |
| **27** | 5,3 | 38 | 19 | 6 | 28 | 1 | 1 | 1 | 0 | 1 | 1 | 1 | 1 | 7 |
| **28** | 3,1 | 28 | 5 | 0 | 7 | 0 | 1 | 1 | 0 | 0 | 0 | 0 | 0 | 2 |
| **29** | 11,5 | 31 | 7 | 0 | 9 | 0 | 0 | 0 | 1 | 0 | 0 | 1 | 0 | 2 |
| **30** | 4 | 40 | 7 | 0 | 10 | 0 | 1 | 1 | 0 | 1 | 0 | 1 | 0 | 4 |
| **31** | 3,9 | 29 | 13 | 2 | 16 | 1 | 1 | 1 | 0 | 1 | 1 | 1 | 0 | 6 |
| **32** | 8,5 | 38 | 6 | 1 | 9 | 1 | 1 | 1 | 1 | 0 | 0 | 0 | 0 | 4 |
| **33** | 7,5 | 32 | 7 | 0 | 9 | 0 | 0 | 1 | 0 | 0 | 0 | 0 | 0 | 1 |
| **34** | 8,4 | 28 | 8 | 0 | 9 | 1 | 1 | 1 | 1 | 1 | 0 | 1 | 0 | 6 |
| **35** | 12,4 | 34 | 12 | 4 | 17 | 1 | 1 | 1 | 1 | 1 | 1 | 1 | 0 | 7 |
| **36** | 3 | 28 | 9 | 0 | 9 | 0 | 1 | 1 | 0 | 0 | 0 | 0 | 0 | 2 |
| **37** | 12 | 40 | 6,5 | 1 | 8,5 | 1 | 1 | 1 | 1 | 1 | 1 | 1 | 1 | 8 |
| **38** | 5,8 | 27 | 10,5 | 1 | 14,5 | 1 | 1 | 1 | 1 | 0 | 0 | 1 | 0 | 5 |
| **39** | 7,8 | 34 | 19 | 4 | 26 | 1 | 1 | 1 | 1 | 1 | 1 | 1 | 1 | 8 |
| **40** | 19,7 | 35 | 3,5 | 0 | 3,5 | 0 | 0 | 0 | 0 | 0 | 0 | 0 | 0 | 0 |
| **41** | 3 | 34 | 13 | 4 | 20 | 0 | 0 | 0 | 0 | 0 | 0 | 0 | 0 | 0 |
| **42** | 2,1 | 25 | 13 | 0 | 14 | 1 | 1 | 1 | 1 | 1 | 0 | 1 | 1 | 7 |
| **43** | 9,2 | 27 | 12,5 | 4 | 18,5 | 1 | 1 | 1 | 1 | 1 | 1 | 1 | 1 | 8 |
| **44** | 3 | 29 | 11 | 4 | 18 | 1 | 1 | 1 | 1 | 1 | 1 | 1 | 1 | 8 |
| **45** | 14,4 | 33 | 12,5 | 2 | 15,5 | 1 | 1 | 1 | 1 | 1 | 1 | 1 | 1 | 8 |
| **46** | 6,7 | 35 | 14 | 2 | 17 | 1 | 1 | 1 | 1 | 1 | 1 | 1 | 1 | 8 |
| **47** | 8,3 | 29 | 13,5 | 4 | 20,5 | 1 | 1 | 1 | 0 | 0 | 0 | 1 | 0 | 4 |
| **48** | 3 | 30 | 12 | 4 | 18 | 0 | 0 | 1 | 0 | 1 | 1 | 0 | 0 | 3 |
| **49** | 13,2 | 40 | 10 | 0 | 13 | 0 | 1 | 1 | 0 | 1 | 0 | 1 | 1 | 5 |
| **50** | 3 | 28 | 9 | 0 | 10 | 0 | 1 | 1 | 0 | 0 | 1 | 0 | 0 | 3 |
| **51** | 3 | 40 | 13 | 4 | 19 | 1 | 1 | 1 | 0 | 0 | 0 | 0 | 0 | 3 |
| **52** | 3 | 34 | 14,5 | 0 | 16,5 | 0 | 0 | 0 | 0 | 0 | 0 | 0 | 0 | 0 |
| **53** | 3 | 26 | 15,5 | 4 | 21,5 | 1 | 1 | 1 | 0 | 0 | 0 | 0 | 0 | 3 |
| **54** | 8,4 | 36 | 16 | 0 | 19 | 0 | 0 | 0 | 0 | 0 | 1 | 0 | 0 | 1 |
| **55** | 13,8 | 25 | 11 | 2 | 15 | 1 | 1 | 1 | 1 | 1 | 1 | 1 | 0 | 7 |
| **56** | 3,6 | 34 | 13 | 5 | 19 | 1 | 1 | 1 | 1 | 1 | 0 | 1 | 1 | 7 |
| **57** | 3,5 | 38 | 13,5 | 4 | 20,5 | 1 | 1 | 1 | 1 | 1 | 1 | 1 | 1 | 8 |
| **58** | 7 | 29 | 7 | 0 | 9 | 1 | 0 | 1 | 0 | 0 | 0 | 0 | 0 | 2 |
| **59** | 3,5 | 31 | 13 | 4 | 20 | 1 | 1 | 1 | 1 | 1 | 1 | 1 | 1 | 8 |
| **60** | 3,2 | 28 | 10 | 0 | 10 | 0 | 0 | 0 | 0 | 0 | 0 | 0 | 0 | 0 |
| **61** | 7 | 34 | 10,5 | 2 | 13,5 | 1 | 0 | 1 | 0 | 1 | 0 | 1 | 0 | 4 |
| **62** | 3 | 30 | 15 | 4 | 20 | 1 | 1 | 1 | 0 | 1 | 0 | 1 | 1 | 6 |
| **63** | 7,6 | 31 | 13 | 4 | 19 | 0 | 1 | 1 | 0 | 1 | 1 | 1 | 1 | 6 |
| **64** | 9 | n.a. | 8,5 | 1 | 11,5 | 1 | 1 | 1 | 1 | 1 | 0 | 1 | 0 | 6 |
| **65** | 7,3 | 35 | 17,5 | 2 | 20,5 | 1 | 1 | 1 | 0 | 1 | 0 | 1 | 1 | 6 |
| **66** | 4,1 | 33 | 14 | 4 | 21 | 1 | 1 | 1 | 0 | 1 | 0 | 1 | 1 | 6 |
| **67** | 3 | 37 | 13,5 | 2 | 18,5 | 0 | 0 | 1 | 0 | 1 | 1 | 1 | 1 | 5 |
| **68** | 4,2 | 39 | 12 | 0 | 12 | 0 | 1 | 1 | 0 | 1 | 1 | 1 | 1 | 6 |
| **69** | 12,8 | 35 | 13 | 0 | 14 | 1 | 1 | 1 | 1 | 1 | 0 | 1 | 1 | 7 |
| **70** | 4,7 | 24 | 16 | 4 | 23 | 1 | 1 | 1 | 1 | 1 | 0 | 1 | 1 | 7 |
| **71** | 3 | 32 | 16,5 | 4 | 23,5 | 1 | 1 | 1 | 0 | 1 | 1 | 1 | 1 | 7 |
| **72** | 3 | 32 | 15 | 0 | 18 | 1 | 1 | 1 | 1 | 1 | 0 | 1 | 1 | 7 |

**GA**= Gestational Age; **n.a.=** not available; **H**=Hemispheric; **S**= subcortical; **G**=global; **V**= Visual; **VTS**=Visual Total Score
